# Suppression of Soft Tissue Sarcoma Growth by a Host Defense-Like Lytic Peptide

**DOI:** 10.1371/journal.pone.0018321

**Published:** 2011-03-31

**Authors:** Lars Steinstraesser, Jennifer Hauk, Cornelius Schubert, Sammy Al-Benna, Ingo Stricker, Hanns Hatt, Yechiel Shai, Hans-Ulrich Steinau, Frank Jacobsen

**Affiliations:** 1 Laboratory for Wound Healing and Molecular Oncology, Department of Plastic Surgery, BG University Hospital Bergmannsheil, Ruhr University Bochum, Bochum, North Rhine-Westphalia, Germany; 2 Department of Pathology, BG University Hospital Bergmannsheil, Ruhr University Bochum, Bochum, North Rhine-Westphalia, Germany; 3 Department of Cell Physiology, Ruhr University Bochum, Bochum, North Rhine-Westphalia, Germany; 4 Department of Biological Chemistry, Weizmann Institute of Science, Rehovot, Israel; Florida International University, United States of America

## Abstract

**Background:**

Soft tissue sarcoma (STS) is an anatomically and histologically heterogeneous neoplasia that shares a putative mesenchymal cell origin. The treatment with common chemotherapeutics is still unsatisfying because of association with poor response rates. Although evidence is accumulating for potent oncolytic activity of host defense peptides (HDPs), their potential therapeutic use is often limited by poor bioavailability and inactivation in serum. Therefore, we tested the designer host defense-like lytic D,L-amino acid peptide [D]-K_3_H_3_L_9_ on two STS cell lines *in vitro* and also in an athymic and syngeneic mouse model. In recent studies the peptide could show selectivity against prostate carcinoma cells and also an active state in serum.

**Methods:**

*In vitro* the human synovial sarcoma cell line SW982, the murine fibrosarcoma cell line BFS-1 and primary human fibroblasts as a control were exposed to [D]-K_3_H_3_L_9_, a 15mer D,L-amino acid designer HDP. Cell vitality in physiological and acidic conditions (MTT-assay), cell growth (BrdU) and DNA-fragmentation (TUNEL) were investigated. Membrane damage at different time points could be analyzed with LDH assay. An antibody against the tested peptide and recordings using scanning electron microscopy could give an inside in the mode of action. *In vivo* [D]-K_3_H_3_L_9_ was administered intratumorally in an athymic and syngeneic (immunocompetent) mouse model with SW982 and BFS-1 cells, respectively. After three weeks tumor sections were histologically analyzed.

**Results:**

The peptide exerts rapid and high significant cytotoxicity and antiproliferating activity against the malignant cell lines, apparently via a membrane disrupting mode of action. The local intratumoral administration of [D]-K_3_H_3_L_9_ in the athymic and syngeneic mice models significantly inhibited tumor progression. The histological analyses of the tumor sections revealed a significant antiproliferative, antiangiogenic activity of the treatment group.

**Conclusion:**

These findings demonstrate the *in vitro* and *in vivo* oncolytic activity of [D]-K_3_H_3_L_9_ in athymic and syngeneic mouse models.

## Introduction

Soft tissue sarcomas (STS) are a group of histologically and genetically diverse neoplasms that account for approximately 1% of all adult malignancies [1]. STS are of putative mesenchymal derivation and can involve connective tissue structures as well as viscera and integument anywhere in the human body [1]. Overall survival is approximately 50% at 5 years [2] and up to 60% of high grade STS develop distant metastases [3]; these data support the use of an aggressive approach for the treatment STS. Effective treatment usually requires surgical ablation in combination with radiotherapy and/or chemotherapy as the clinical gold standard and could improve the local control rates [4]. In spite of this promising therapeutic treatment local relapse still occurs in up to 22% of the patients [5], [6]. Unfortunately, late diagnosis often results in distant metastases primarily via the hematogenous route and particularly to the lungs (70–80%) [7].

Chemotherapeutic drugs damage malignant cells by a variety of mechanisms (e.g., DNA cleavage/alkylation and topoisomerase II inhibition) that are eventually translated into apoptotic signals. Unfortunately, STS does not respond well to single or multiple drug regimens [8]. In addition, chemical oncolytic agents are nonspecific and, consequently, damage healthy tissues as well. This has stimulated the search for new oncolytic agents with new modes of action and with a potential to overcome the inherent resistance [9], [10].

Host defense peptides (HDPs) have aroused interest as potential oncolytic agents that overcome the limits of current drugs [10], [11]. As part of the innate immune system, HDPs are expressed in nearly every kind of organism, from plants over amphibians to mammals [10].

Initially classified as exclusively antimicrobial active substances, HDPs have demonstrated significant cytotoxic effects against a wide range of malignant cells including e.g. melanoma, breast- and lung cancer [10], [12], [13]. The oncolytic effect of HDPs depends on their amphipathic, cationic structure [14]. The positive charge of the peptides is proposed to initiate electrostatic interaction with the negatively charged membrane of tumor cells which could lead to permeation of the peptide into the membrane and a subsequent complete membrane disruption [10], [14], [15], [16], [17].

Despite the potent oncolytic activity of these peptides *in vitro*, studies *in vivo* are very limited, mainly because of their inactivation in serum, partially because of their binding to serum components and their enzymatic degradation. This has led to the development of synthetic D-amino acid analogues. D-amino acid peptides could show potent oncolytic activity and high selectivity in prostate carcinoma and preserved their activity also in xenograft models *in vivo* [18], [19]. The differences between naturally occurring lytic L- compared to artificially synthesized D-amino acid peptides is their modified structure. While in solution both peptides show unordered structure characteristics, in and on membranes they have different behaviors. L-amino acids often assume α-helical structures. D-amino acids instead can bind better to negatively charged membranes of tumors than to zwitterionic membranes of normal mammalian cells [19]. Another important point is the pH dependence of HDPs. Frequently tumor tissue possesses an acidic extracellular milieu because of the disordered metabolic and nutritional environment [20], [21], [22]. Much current research is focused on designing HDPs which are only active in acidic environment, as this may be promising feature in oncolytic treatment. The host defense-like lytic peptide [D]-K_3_H_3_L_9_ used in this study is composed of the D- and L-amino acids lysine, histidine and leucin. Histidine (pKa, ∼6.1) is protonated below pH 7 [23], [24]. The acidic environment created by solid tumors activates the HDP by making it cationic. Therefore selectivity against malignant tissue is achieved. In previous studies the [D]-K_3_H_3_L_9_ peptide has already shown an oncolytic activity against prostate carcinoma in cell culture and also in an *in vivo* xenograft model [23].

The aim of this study was to assess the oncolytic activity of the D-amino acid peptide [D]-K_3_H_3_L_9_ in an athymic and immunocompetent model and to analyze the potential of this peptide as a therapeutic option against STS [25].

## Materials and Methods

### Ethics statement

#### Human ethics

The participants gave their written informed consent, and the study was reviewed and approved by the ethical committee of the BG University Hospital Bergmannsheil, Ruhr-University Bochum, Germany with the permit number 2353.

#### Animal ethics

The treated animals were congenitally athymic nude mice (Foxn1nu/nu) (Harlan Winkelmann GmbH, Borchen, Germany) and immunocompetent C57BL/6 mice, 5–6 weeks old, weighing about 20–25 g. They were housed in ventilated, pathogen-free racks under a 12 h light-dark photoperiodicity with controlled humidity and temperature (23±2°C). Sterility was maintained during all surgical procedures.

The Animal Care and Use Committee at the district government of North Rhine Westphalia in Arnsberg, Germany approved all animal experiments with the permit numbers 9.93.2.10.32.07.026 and 8.87-50.10.37.09.235. It was conducted in compliance with the Guide for the Care and Use of Laboratory Animals' from the German Animal Welfare Act, which conforms to the provisions of the Declaration of Helsinki in 1995.

### Cell culture

The SW982 human synovial sarcoma cell line (Cell Line Service (CLS), Eppelheim, Germany), last authenticated via nonaplex PCR in July, 2008) was grown in DMEM supplemented with 10% FCS (Thermo Fisher Scientific Inc., Waltham, MA, USA) and 1% penicillin/streptomycin (PAA laboratories, Pasching, Austria). The murine fibrosarcoma cell line BFS-1wt (generously provided by Prof. T. Hehlgans, Department of Immunology, Regensburg, Germany) were maintained in RPMI 1640 supplemented with 10% FCS and antibiotics. The primary human fibroblasts were isolated from patients' tissue at the BG University Hospital Bergmannsheil. The study was approved by the local ethics committee, and all of the patients gave written informed consent. For preparation of the freshly received human skin dermis was separated from epidermis. Primary human fibroblasts were obtained as previously described [26]. All cells were cultured at 37°C in a humified atmosphere of 5% CO_2_. Before starting the experiments, cells were grown to confluence, changing medium twice a week.

### Peptide synthesis and purification

The host defense-like lytic peptide [D]-K_3_H_3_L_9_ was synthesized by a solid-phase method and purified by RP-HPLC (NeoMPS S.A., Strasbourg, France). The sequence of the peptide was a generous gift of Prof. Yechiel Shai, Department of Biological Chemistry, The Weizmann Institute of Science, Rehovot, Israel. [D]-K_3_H_3_L_9_ is a short 15-mer D,L-amino acid peptide analogue (LHLLHKLLKHLLKLL-NH_2_, underlined letters are D-amino acids).

### Cell vitality

The metabolic activity was measured via MTT assay according to the standard protocol. Short: Cells were seeded in 96-well microtiter plates (Corning Inc., New York, NY, USA) in a concentration of 1×10^4^ cells per well. The following day, cells were incubated in FCS-free media with different concentrations of [D]-K_3_H_3_L_9_ (0–100 µM) for 24 hours. Thereafter the cells were incubated in fresh media containing Thiazolyl Blue Tetrazolium Bromid (MTT) solution for another 4 hours. Vital cells integrated the dye as a sign of active metabolism. Dimethyl sulfoxide (DMSO) (Carl Roth GmbH, Karlsruhe, Germany) and glycine buffer (containing 0.1M glycine, 0.1M NaCl, pH 10.5, adjusted with NaOH) were added to the wells. The amount of integrated dye represented the level of metabolism and was quantified at 562 nm by an Elx808 Ultra Microplate Reader (Bio-Tek Instruments GmbH, Bad Friedrichshall, Germany). The LD_50_ for each cell type was obtained from the dose-dependent cell viability curves. The test was carried out at physiological and acidic conditions (pH 7.3 and pH 6.0).

### Cell proliferation

The cell proliferation was measured via BrdU cell proliferation enzyme-linked immunosorbent assay (ELISA) kit (Roche Diagnostics GmbH, Mannheim, Germany) according to the manufacturer's instructions. Briefly, cells were seeded at 3×10^4^ cells/well in 96-well plates, grown for 24 hours and then incubated with fresh FCS-free medium containing the host defense-like lytic peptide [D]-K_3_H_3_L_9_ (0–100 µM) for 24 hours.

BrdU labeling solution was added and incubated for another 22 hours. Proliferating cells integrated BrdU, a pyrimidine analogue, into their DNA. The level of proliferation was quantified by the light emission detected via an Orion microplate luminometer (Berthold Detection Systems, Pforzheim, Germany). LD_50_ values for each cell type were evaluated. Cell proliferation was determined in triplicate. The results are expressed as a percentage of the cell viability in comparison to the negative control.

### DNA fragmentation

Cells undergoing DNA-cleavage were detected via a terminal deoxynucleotidyl transferase (TdT)-mediated 2′-deoxyuridine 5′-triphosphate (dUTP) nick-end labeling (TUNEL) assay. Cells were seeded on chamber slides (Nalge Nunc International, Naperville, IL, USA) at 35,000 cells per chamber, grown for 24 hours and then incubated with [D]-K_3_H_3_L_9_ (0 µM–62.5 µM) for 24 h. After fixation with 4% formaldehyde solution (Carl Roth GmbH, Karlsruhe, Germany) cells were treated with TdT (Roche Diagnostics GmbH, Mannheim, Germany). Slides treated with DNase I (Qiagen Incorporated, Valencia, CA, USA) served as positive controls. Slides treated without TdT served as negative controls. TdT catalyzed the addition of fluorescent dUTP (Invitrogen, Eugene,OR, USA) to DNA fragments caused by DNA-cleavage. Cells labeled with dUTP were detected and evaluated microscopically via a Zeiss Axioskop 2 Plus microscope (Carl Zeiss Jena GmbH, Jena, Germany). All experiments were performed in triplicate. Pictures were taken using the software Zeiss Axio Vision 3.1. (Carl Zeiss Jena GmbH, Jena, Germany).

### Membrane integrity

The membrane integrity of the cells after incubation with [D]-K_3_H_3_L_9_ was measured by LDH release. The used CytoTox 96® Non-Radioactive Cytotoxicity assay (Promega, Madison, WI, USA) was done parallel to the MTT assay at different time points (2 min, 15 min, 30 min, 2 h, 4 h, 24 h) and after incubation with the peptide at 1, 2 and 4 multiples of the LD_50_ values. The kinetic rate for cytotoxicity was monitored concomitantly with the time dependence for leakage of intracellular lactate dehydrogenase (LDH) as follows: after incubation with the peptide, 50 µl of the supernatants was collected and transferred to new 96-well plates. LDH-solution was added for 30 min. After stopping the reaction the LDH release was measured at 490 nm by an Elx808 Ultra Microplate Reader (Bio-Tek Instruments GmbH, Bad Friedrichshall, Germany). The remaining medium was immediately replaced with fresh medium and cytotoxicity was determined by MTT assay (see Cell vitality).

### Localization analysis

Cells (35.000 cells/chamber) were seeded and grown for 24 hours on chamber slides (Nalge Nunc International, Naperville, IL, USA). After incubation with [D]-K_3_H_3_L_9_ at different time points (30 min, 4 h and 24 h) cells were fixed and treated with citrate buffer for high temperature antigen retrieval. Blocking buffer was used to avoid non specific binding. Rabbit-anti-[D]-K_3_H_3_L_9_ antibody (Polypeptide Laboratories, Strasbourg, France) was applied to detect the position of the peptide within the cells. After incubation with a biotinylated anti-rabbit antibody and Streptavidin Alexa Fluor 488, nuclei were counterstained with DAPI (Invitrogen Corporation, Carlsbad, CA, USA). Results were evaluated microscopically by a Zeiss LSM-510 Meta confocal laser scanning microscope system (Carl Zeiss Jena GmbH, Jena, Germany).

### Membrane visualization

Cells (10.000 cells/cover glass) were seeded on sterile cover glasses (12 mm in diameter, NeoLab, Heidelberg, Germany). After 24 h cells were incubated with a lethal dose (LD_100_, here: 50 µM) of the [D]-K_3_H_3_L_9_ peptide or with PBS as a control for different time points (30 min, 4 h, 24 h). Subsequently, cells were washed with PBS and fixed in 3.7% glutardialdehyde, following dehydration through an ascending alcohol series (50%, 70%, 90%, 100% EtOH, 5 min/step). After drying samples were coated with gold particles and analyzed via a scanning electron microscope (DSM 982, Carl Zeiss, Oberkochen, Germany).

### Hemolytic activity

The hemolytic activity of the [D]-K_3_H_3_L_9_ peptide was determined on human as well as murine erythrocytes. Therefore, whole blood was removed and transferred into EDTA tubes. After dilution of 5 ml blood in NaCl solution the suspension was centrifuged by 1200 g without break for 5 min. The supernatant (plasma) was discarded and erythrocyte suspension was washed with NaCl until the supernatant was clear. A 2.8% (v/v) erythrocyte suspension was prepared and incubated with different concentrations of the peptide (0–100 µM) for different length of time (30 min, 2 h, 24 h). The absorbance of the supernatants was measured at 540 nm by an ELISA plate reader Elx808 Ultra Microplate Reader (Bio-Tek Instruments GmbH, Bad Friedrichshall, Germany). Correlating the measured values of treated (A_sample_) and untreated (A_control_) erythrocytes led to the percentage of hemolytic activity. Total hemolysis (A_total_) was obtained by treating erythrocytes with 0.1% Triton-X100 (Merck, Darmstadt, Germany).
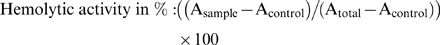



### Solid tumor model

Human synovial sarcoma SW982 and murine fibrosarcoma BSF-1 cells in Matrigel (BD Biosciences, San Jose, CA, USA) were injected subcutaneously (1×10^6^ cells) into the animal's flank. Every second day mice were weighed and tumor volume was determined with the formula of length×width×depth×0.5 in mm^3^. When the tumor volume reached an averaged volume of 138 mm^3^ (SW982) and 130 mm^3^ (BFS-1) the animals were grouped according to the principles of a randomized control trial. [D]-K_3_H_3_L_9_ was injected intratumorally three times per week for three weeks (single dose 8.5 mg/kg, SW982 n = 7, BFS-1 n = 6). Injection sites varied. PBS (pH 7.0–7.5) was applied as control substance (SW982 n = 5, BFS-1 n = 4). Animals were sacrificed and tumors were analyzed.

To verify the success of therapy a long-term experiment was carried out. Mice were treated with the [D]-K_3_H_3_L_9_ peptide (n = 10) or PBS as a control (n = 5) as described above. An observation of tumor size followed over a period of six weeks. After reaching a tumor length of over 20 mm the experiment was terminated and the animals were euthanized. The evaluation of the data was based on a Kaplan-Meier graph.

### Histologic and immunofluorescent staining

Excised tumors were fixed in 5% buffered formaldehyde. Paraffin-embedded 2–3 µm sections were stained with haematoxylin and eosin (H&E). Slides of tumors treated with [D]-K_3_H_3_L_9_ or with PBS were examined under a microscope (Zeiss Axioskop 2 Plus microscope) and photographed (AxioCam HRc). For immunohistological examination slides were deparaffinized and rehydrated. High temperature antigen retrieval in citrate buffer (Vector Laboratories, Burlingame, CA, USA) was performed followed by incubation with blocking buffers to avoid nonspecific binding of antibodies. Tissue sections were incubated with monoclonal anti-human Ki67 antibody (Acris Antibodies GmbH, Herford, Germany) specific to proliferating cells, anti-Laminin antibody (Sigma-Aldrich, St. Louis, MO, USA) specific to the basal lamina as can be found in blood vessels and anti-CD3 antibody (Acris Antibodies GmbH, Herford, Germany) specific to T-cells. After incubation with a biotinylated secondary anti rabbit antibody, Streptavidin Alexa Fluor 488 conjugate was added. Nuclei were counterstained with the fluorescence DNA dye DAPI. Results were evaluated microscopically via a Zeiss Axioskop 2 Plus microscope. Pictures were taken using an AxioCam HRc and the software Zeiss Axio Vision 3.1. For quantitative analysis Ki67-positive cells were counted. Therefore 10 high power fields (HPFs) (magnification: 400×) of non-necrotic areas of four different tumors from each group were assessed by three independent investigators.

## Results

### *In vitro* activity of [D]-K_3_H_3_L_9_ at acidic and physiological pH

**Figure 1:**
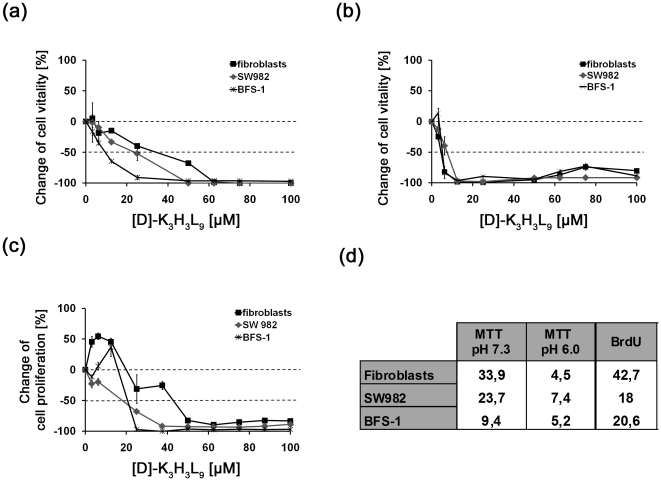
Impact of cell metabolism and proliferation of human synovial sarcoma (SW982) and murine (BFS-1) cell line after incubation with increasing concentrations of [D]-K_3_H_3_L_9_. (a) Metabolic activity of the cells in physiological conditions (pH 7.3). (b) Metabolic activity of the cells in an acidic milieu (pH 6.3). The MTT-assay revealed dose-dependendent cytotoxic activity in both cases. The cell vitality was significantly (p<0.05) lower in an acidic milieu compared to physiological conditions. (c) [D]-K_3_H_3_L_9_ displayed an anti-proliferative effect towards both sarcoma cell lines. (d) Lethal dose (LD_50_ in µM) of human synovial sarcoma (SW982) and murine fibrosarcoma (BFS-1) cell line after incubation with [D]-K_3_H_3_L_9_.

The viability of the cells after treatment with [D]-K_3_H_3_L_9_ was determined at physiological (pH 7.3) and acidic (pH 6.0) conditions with the MTT assay. A disordered metabolic and nutritional situation and an insufficient vascularisation often result in an acidic extracellular environment in malignant tumor tissue. Fig. 1 (a+b) shows the metabolic activity of the cells after incubation with different concentrations of the peptide. In physiological conditions (a) [D]-K_3_H_3_L_9_ shows a tendency for selective activity against malignant cells compared to normal primary fibroblasts. Note that the metabolic activity was significantly (p<0.05) lower in an acidic milieu (b) compared to physiological conditions in all tested cell lines, also in primary fibroblasts. A comparison between primary human fibroblasts in physiological pH, where they normally occur, and tumor cells in acidic milieu, demonstrates the selectivity of [D]-K_3_H_3_L_9_ against malignant cells. The LD_50_ of the tested sarcoma cells in acidic milieu is 7.4 fold and 5.2 fold lower than that of the fibroblasts in physiological condition for SW982 and BFS-1 cells, respectively.

### Decrease of cell proliferation

The proliferation of the cells was measured by BrdU incorporation into DNA (Fig. 1 (c)). Note that fibroblasts and also the murine fibrosarcoma cell line BFS-1 show an increase of proliferation after incubation with low concentration of the peptide. At higher dosage [D]-K_3_H_3_L_9_ displayed an antiproliferative effect towards both sarcoma cell lines. Noteworthy is the fact that primary human fibroblasts need a 2 fold higher dosage for reaching their LD_50_ value of 42.7 µM (Fig. 1(d)).

### DNA-fragmentation after [D]-K_3_H_3_L_9_ treatment

**Figure 2:**
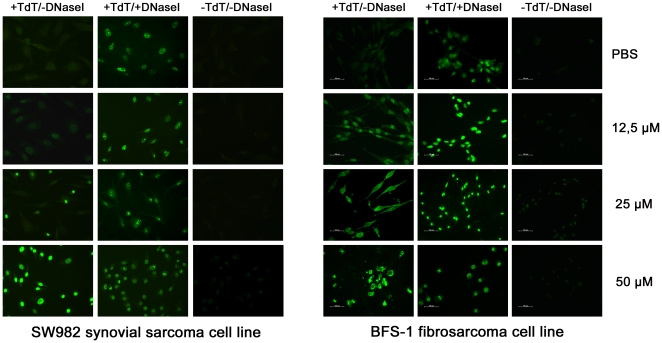
DNA-fragmentation (TUNEL assay) after treatment with different concentrations (12,5 µM, 25 µM, 50 µM) of the peptide and PBS as a control. An apoptotic effect of [D]-K_3_H_3_L_9_ could already be detected at a peptide concentration of 25 µM and 12.5 µM in SW982 and BFS-1 cells, respectively. In the positive control the cells underwent DNA-cleavage after incubation with DNase I and were therefore labeled with dUTPs (green). Negative controls received no treatment with the labeling enzyme TdT.

The TUNEL assay allows the analysis of potential DNA-cleavage, one of the results of apoptosis. Fig. 2 shows both tested cell lines after incubation with different concentrations of the peptide. As well as for the determination of cell vitality and proliferation the assay revealed a dose dependence regarding the genotoxic effect of [D]-K_3_H_3_L_9_. At a concentration of 25 µM the synovial sarcoma cells (SW982) began to show DNA-fragmentation, whereas the fibrosarcoma cells (BFS-1) already showed an effect at 12.5 µM. At a peptide concentration of 50 µM almost all sarcoma cells could show DNA-cleavage. Note that the BFS-1 cells show a destroyed structure after treatment with 50 µM of [D]-K_3_H_3_L_9_ whereas SW982 cells have almost intact cell compartments and membranes.

### Time dependent membrane disruption

**Figure 3:**
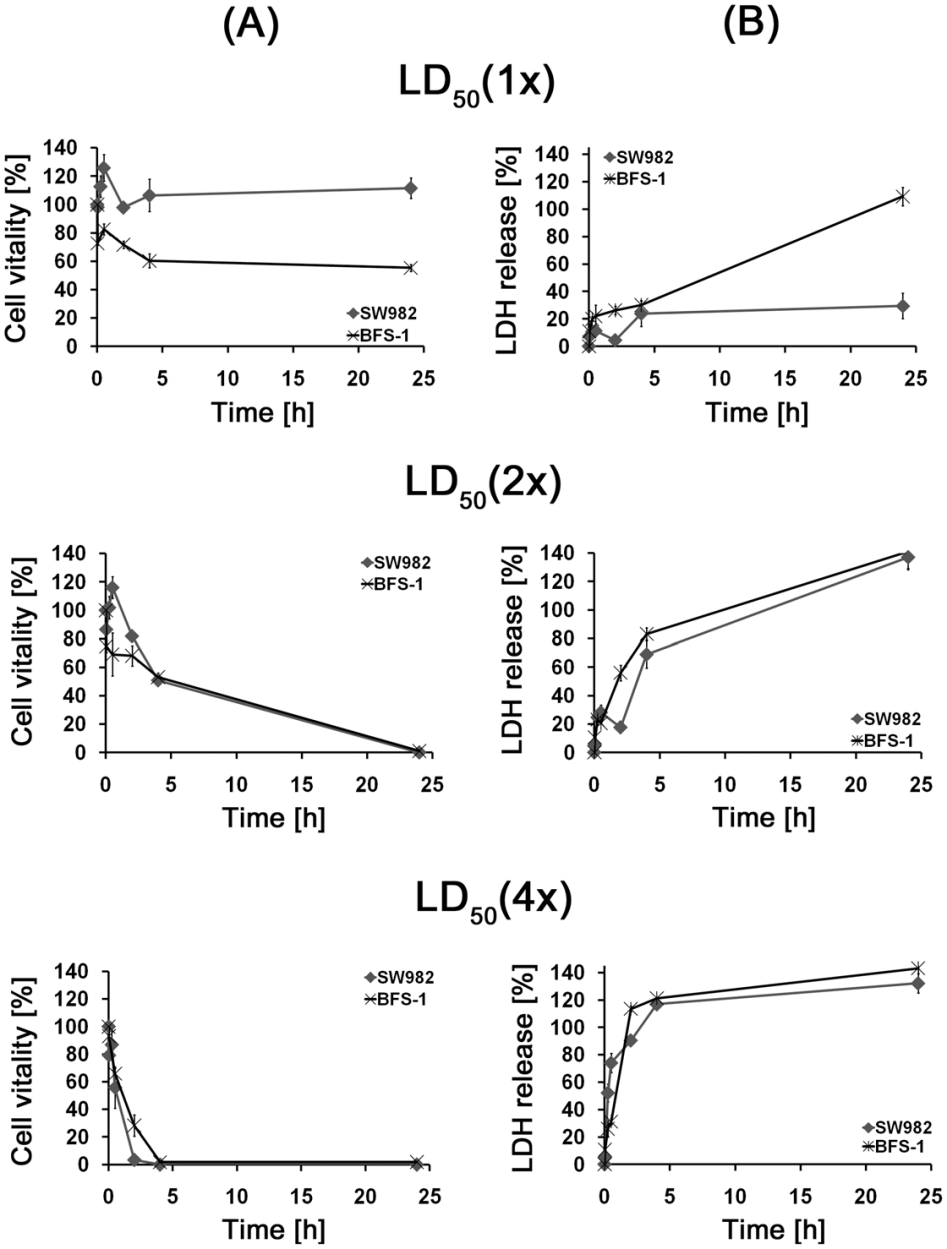
Parallel testing of (a) cell vitality with MTT- and (b) membrane disruption with the LDH-assay at different time points (2 min, 15 min, 30 min, 2 h, 4 h, 24 h) and concentrations (LD_50_: 1×, 2×, 4×) of [D]-K_3_H_3_L_9_. The membrane integrity of the cells after incubation with [D]-K_3_H_3_L_9_ at the LD_50_ in 2 and 4 multiples showed a parabolic curve progression with over hundred percent LDH release after 24 h. The LDH release appears to rise faster than the cell viability decreases. These results indicate that the destruction of membrane integrity causes the decreased cell vitality after treatment with the peptide.

The parallel testing of cell vitality with MTT and membrane disruption with the LDH assay at different time points can give a hint of the mode of action of [D]-K_3_H_3_L_9_. Sarcoma cells were treated with the previously determined LD_50_ of the peptide in 1, 2 and 4 multiples. Fig. 3 shows the viability of the sarcoma cell lines (A) compared to the LDH release (B).

At a concentration of simple LD_50_ the synovial sarcoma cell line SW982 did not show any limitation in cell vitality. The cells also showed only a slight increase of the LDH release. Even after 24 hour incubation the release only increases to a value of 29.5 µM. The BFS-1 cells revealed a low decrease of vitality and increase of LDH. Only after one day of incubation nearly all cells have lost their membrane integrity. Note that still half of the BFS-1 cells are alive after 24 h. The membrane integrity of the cells after incubation with [D]-K_3_H_3_L_9_ at the LD_50_ in 2 multiples showed a parabolic curve progression with over hundred percent LDH release after 24 h. The metabolic activity of the cells decreased more slowly. After 4 h incubation only half of the cells (SW982: 50.9%, BFS-1: 53.2%) showed an inhibition of the metabolic activity. After 24 h incubation the metabolism of the cells was completely inhibited. Incubation of the cells with LD_50_ in 4 multiples showed comparable results. A strong decrease of cell vitality even after low incubation periods is linked to a strong and fast increase of LDH release. Nevertheless, the LDH release appears to rise faster than the vitality decreases. These results indicate that the destruction of membrane integrity causes the decreased cell vitality after treatment with the peptide.

### Cellular localization of [D]-K_3_H_3_L_9_

**Figure 4:**
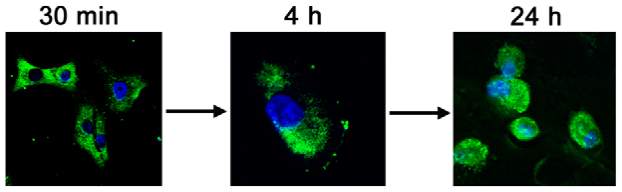
Mode of action analysis with primary antibody against [D]-K_3_H_3_L_9_. Representative images of SW982 cells treated with a lethal dose of the peptide. After 30 minutes the peptide is located in the cytoplasm. The cells still have a healthy structure and cell extensions. After 4 hours of incubation the membranes are destroyed and cell extensions are lost. 24 hour incubation with [D]-K_3_H_3_L_9_ leads to a complete vanish of cytoplasm.

To investigate potential targets of [D]-K_3_H_3_L_9_ cells were treated with a specific antibody against the HDP. Fig. 4 gives a representative example of SW982 cells after incubation with [D]-K_3_H_3_L_9_ at different time points. After 30 min the fluorescent staining indicates a location of the peptide in membrane and even in intracellular components. Note that the cells are in structure, cell extensions are still visible. After 4 hours the cell membrane is disrupted and most cell extensions are withdrawn. The cells changed their morphological shape into a more condensed form. The peptide is still located intracellular, the nuclei are not involved. After 24 hours of incubation the peptide also infiltrates the nuclei and cell membranes are often completely destroyed.

### Membrane modification after incubation with K_3_H_3_L_9_-NH_2_

**Figure 5:**
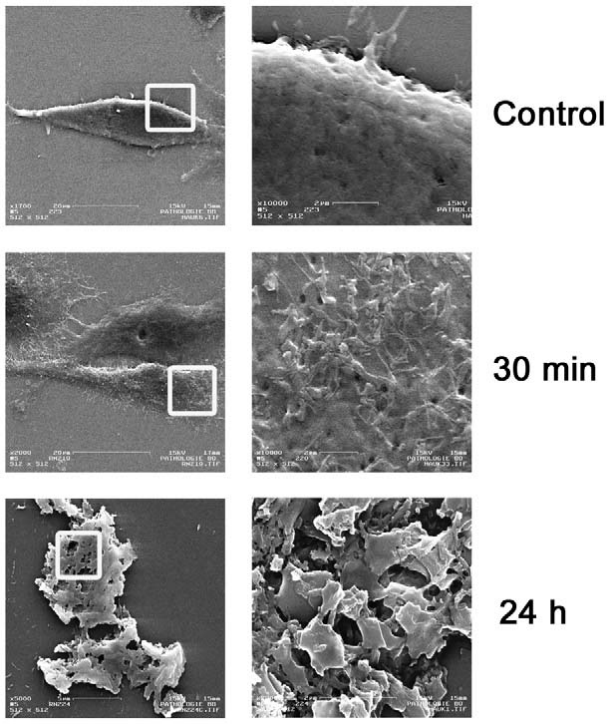
Representative scanning electron microscopy images of SW982 cells treated with [D]-K_3_H_3_L_9_ for 30 min and 24 h. Magnification 2.000× (left column) and 10.000× (right column). Squares represent further magnified region. The figures display a complete membrane disruption after 24 hours.

The scanning electron microscopy images give a hint on the mode of action of [D]- K_3_H_3_L_9_ on plasma cell membranes. Fig. 5 visualizes the differences between control cells and cells treated with [D]- K_3_H_3_L_9_ at indicated time points. Cells treated with PBS are characterized by a smooth cell surface, whereas even after a short incubation time of 30 min the cell membrane is affected significantly which is demonstrated by a starting loss of membrane integrity illustrated by thread-like projections. After 24 h the plasma membrane is completely destroyed.

### Hemolytic activity of [D]-K_3_H_3_L_9_

**Figure 6:**
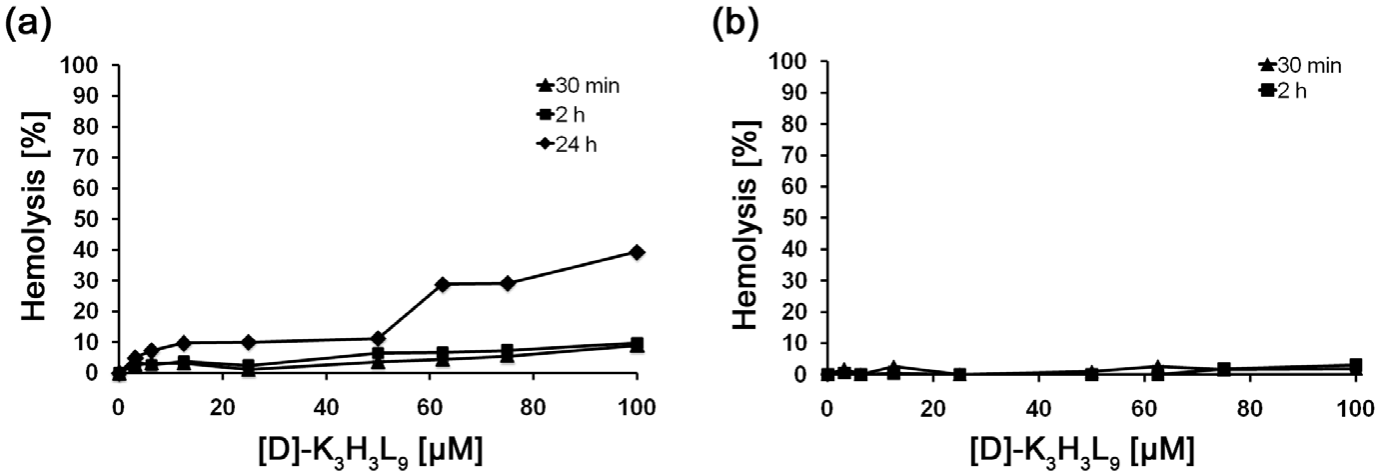
Hemolytic activity of isolated (a) human and (b) murine erythrocytes after incubation with different concentrations (0–100 µM) of the host defense-like lytic peptide [D]-K_3_H_3_L_9_. Even at highest concentration (100 µM) both types of erythrocytes show less than 10% hemolysis after 30 min and 2 hours. After 24 h incubation period human erythrocytes show an increased hemolytic activity up to 39%.

The influence of the peptide against human and murine erythrocytes was measured using the hemolysis assay. Fig. 6 shows the hemolytic activity on human (a) and murine (b) erythrocytes after incubation with different concentrations of [D]-K_3_H_3_L_9_. After incubation times of 30 min and 2 hours both types of erythrocytes show less than 10% hemolysis compared to the positive control, even at highest peptide concentration. A 24 h incubation period with the peptide could show an increase of hemolytic activity in human erythrocytes up to 39%. Murine erythrocytes could not be evaluated after 24 h as the control has already had a strong hemolytic activity.

### Inhibition of solid soft tissue sarcoma growth by intratumoral administration of [D]-K_3_H_3_L_9_

**Figure 7:**
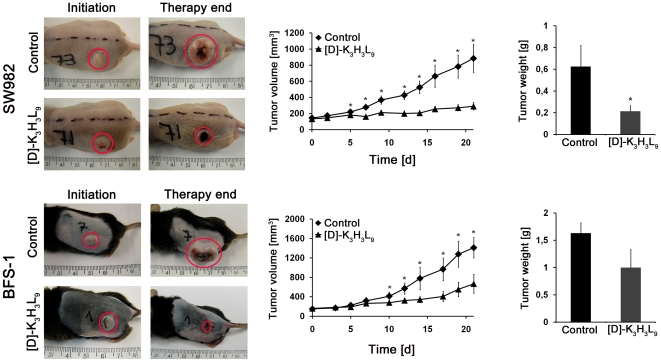
*In vivo* studies. (a) Athymic nude mice model with human synovial sarcoma (SW982) tumor after treatment with PBS (control) and [D]-K_3_H_3_L_9_. (b) After the third day of therapy a significant (* p<0.05) difference in tumor volume of control and peptide treated tumors could be determined (c) Weight of SW982 tumors after three-week treatment with control substance and [D]-K_3_H_3_L_9_. Peptide treated tumors have a significantly (p<0.05) diminished tumor weight (d) Syngeneic, immunocompetent mouse model with murine fibrosarcoma (BFS-1) tumor after treatment with PBS (control) and [D]-K_3_H_3_L_9_ (e) After the fourth day of therapy the tumor volume of peptide treated mice was significantly (p<0.05) diminished compared to control mice (f) Weight of BFS-1 tumors after treatment with control substance and [D]-K_3_H_3_L_9_.

Cells were implanted subcutaneously into the left flank of athymic nude (SW982) or immunocompetent C57BL/6 (BFS-1) mice. After reaching the defined tumor volume the oncolytic activity of [D]-K_3_H_3_L_9_ was assessed. The peptide was injected intratumorally at a dose of 8.5 mg/kg. A significant inhibition of the tumor volume could be reached after therapy in both models. As shown in Fig. 7(a+b;d+e), the control groups of both, athymic and immunocompetent mouse model, displayed exponential tumor growth throughout the three-week therapy. The tumor volume increased to a final mean tumor volume of 886 mm^3^ and 1979 mm^3^ for SW982 and BFS-1 cells, respectively. The local administration of [D]-K_3_H_3_L_9_ leads to a partial remission and in two cases to an almost full remission of the tumor. Already after the third (SW982) and fifth (BFS-1) injection, a significant (p<0.05) difference in tumor growth was observed in both models. The mean tumor volume at the end of the therapy reached a value of 290 mm^3^ and 667 mm^3^ for SW982 and BFS-1 tumors, respectively.

These findings were accompanied by a significant reduction (p<0.05) in dissected tumor weight of the treatment group compared to the control group (Fig. 7 (c)) on the last day of the experiment for SW982 cells. The BFS-1 mouse model demonstrated a tendency (p<0.09) for tumor weight reduction after treatment with the peptide (Fig. 7 (f)).

**Figure 8:**
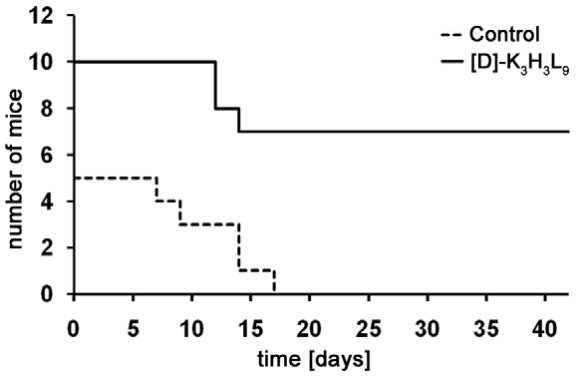
Survival rate of mice after long-term trial. The Kaplan-Meier graph illustrates the differences in overall survival of mice after treatment with [D]-K_3_H_3_L_9_ or PBS as a carrier control. Whereas 100% of the carrier control animals had to be euthanasized before the expire of the 6 weeks-follow-up, 70% of the peptide treated animals survived th e entire period.

The long-term experiment could give an indication of the beneficial effect of the peptide for the period after therapy. We observed the tumor growth up to six weeks after first injection of the peptide. The Kaplan-Meier graph (Fig. 8) illustrates the significant difference between the [D]-K_3_H_3_L_9_ treated animals and the control mice. Whereas 100% of the PBS treated animals had to be euthanized at the latest after 17 days due to excessive tumor growth, 70% of the peptide treated mice survived the entire period. Animals with complete remission of the tumor (2/10 mice) did not show any tumor growth even after the six week-follow-up (data not shown).

**Figure 9:**
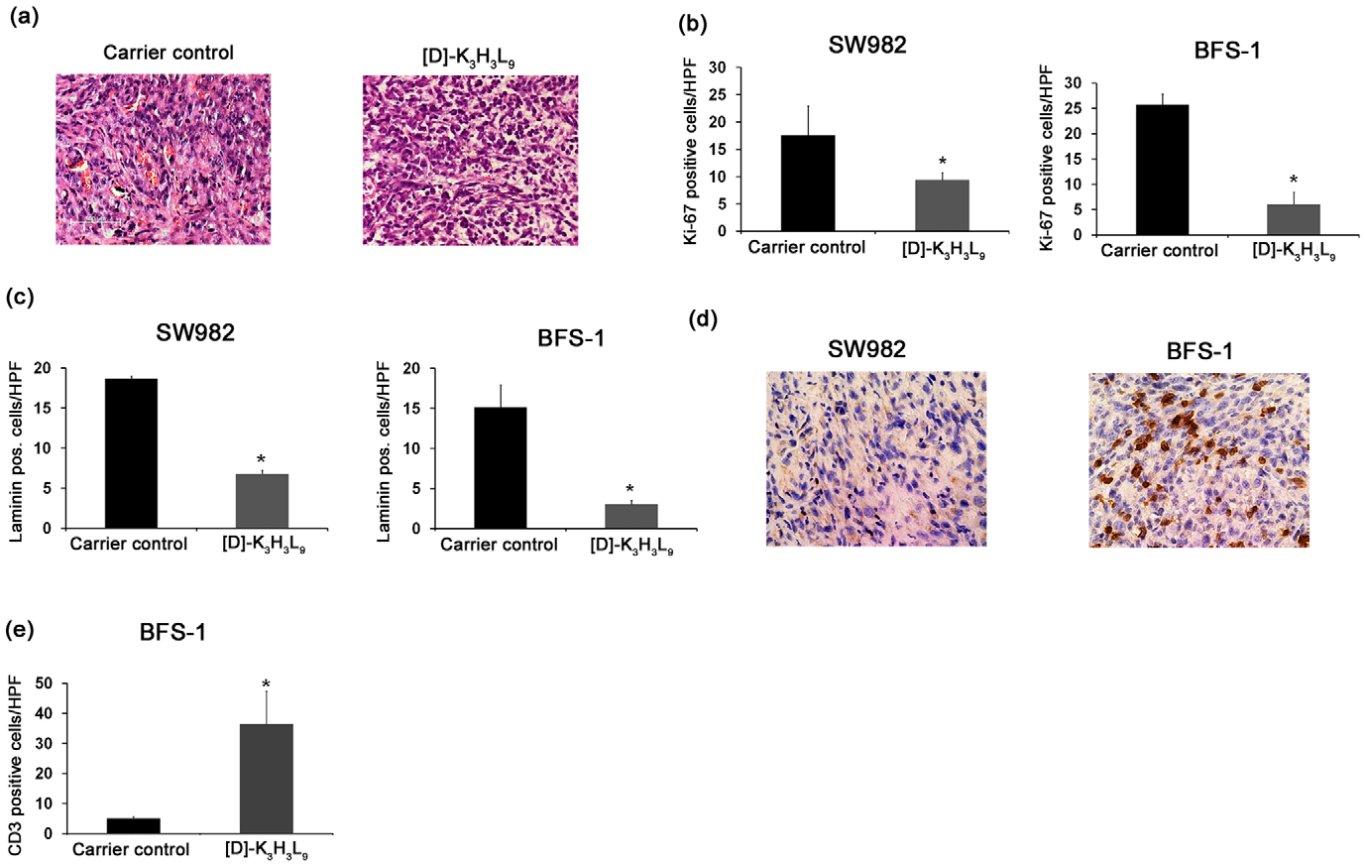
Histological and immunohistological analysis. (a) Exemplary images of H&E stained tumor sections of SW982 cells (200× magnification). The poor nuclear-to cytoplasmic ratio and large spaces between the cells of [D]-K_3_H_3_L_9_ treated tumors indicates a massive cell death. (b) Anti-Ki-67 staining. A significant (p<0.05) reduction of proliferating cells could be detected in [D]-K_3_H_3_L_9_ treated cells compared to the carrier control in both mice models. (c) Anti-laminin staining. Blood vessel density was significantly (p<0.01) diminished in peptide treated tumors compared to the control. (d) Anti-CD3 staining. Compared to the athymic mouse model (SW982) a significant increase (p<0.01) of CD-3 stained T-cells could be determined in [D]-K_3_H_3_L_9_ treated tumors of immunocompetent BFS-1 tumors. (e) Anti-CD3 staining. A comparison between peptide-treated and control-treated tumors in the immune competent BFS-1 model could show a significant increase (p<0.01) of T-cell migration in [D]-K_3_H_3_L_9_ treated tumors.

Histological examination of the tumor was done after resection. Fig. 9 (a) presents two histopathological images (H&E) of representative tumor sections of the synovial sarcoma cell line SW982. In the control xenografts, the tumor contains large numbers of dense, highly proliferative cancer cells. The decrease in tumor staining, a poor nuclear-to-cytoplasmic ratio, and the loose structure in the [D]-K_3_H_3_L_9_ treated mice indicates a massive tumor cell death. This was further verified with Ki67 immunohistochemistry stainings. Fig. 9 (b) shows the results of the HPF-counting of both cell lines. Control tumors had a significant (p<0.05) larger amount of stained cells compared to therapy-treated tumors.

A strong antiangiogenic effect of the peptide could be determined using an anti-laminin-staining. Fig. 9 (c) reflects the HPF-counting of blood vessels after immunhistochemical staining with anti-laminin antibody. In both cases (SW982 and BFS-1) a significant inhibition of vessel formation are seen after [D]-K_3_H_3_L_9_ therapy.

A possible immunomodulatory effect of the peptide was investigated based on an anti-CD3 staining (Fig. 9 (d)).Compared to the athymic mice model the syngeneic BFS-1 model shows a significant increase of T-cells in the tumor environment. Investigating peptide-treated with carrier control-treated tumors in the immunocompetent model could show a significant increase of T-cells migration after [D]-K_3_H_3_L_9_ therapy (Fig. 9 (e)).

## Discussion

The most important result is that [D]-K_3_H_3_L_9_ markedly reduced tumor volume in both athymic and immunocompetent xenografts, and the tumor completely disappeared in two of the mice. The histological evaluation showed necrotic debris in the sections taken from the remaining tumors treated with [D]-K_3_H_3_L_9_, and the density of the cells was very low (Fig. 7b). This is in contrast with the significantly higher amount of mitotic cells in the control tumors. These results demonstrate that the remaining tumor does not proliferate normally. In addition, [D]-K_3_H_3_L_9_ is selective towards and much more active on the sarcoma cells than the primary human fibroblasts.

In STS treatment, designer HDPs may offer an alternative to the low therapeutic indices and toxic side effects of chemotherapeutic agents [11], [27], [28]. The [D]-K_3_H_3_L_9_ used in this study has previously shown dramatic reductions of tumor growth in various human prostate carcinoma xenografts [23]. Solid tumors often have a modified metabolic and nutritional environment compared to normal cells [20]. Fast tumor growth leads to an insufficient vascularisation followed by a diminished wash out of acidic products. In addition, the lack of oxygen contributes to an acidic milieu [21]. The peptide [D]-K_3_H_3_L_9_ consists of lysines, histidines and leucins. Histidine is protonated below pH 7, therefore the peptide should be more cationic at low pH levels. The viability testing in this study revealed an increasing activity of the peptide in acidic environment compared to physiological conditions. A comparison of cell viability between primary human fibroblasts, which normally occur in physiological conditions, at pH 7.3, and malignant cells in acidic milieu (pH 6.3) demonstrates a strong selectivity of [D]-K_3_H_3_L_9_ against cancer cells. The proliferating activity of fibroblasts and also murine fibrosarcoma cells first increased after treatment with low concentrations of the peptide. This could be due to an increased cell metabolism and additional energy expenditure to transfer molecules across the cell membrane in the extracellular matrix.

Because most chemotherapeutics act by damaging DNA or by interfering with DNA replication, they need to enter the cell to be functional [29]. Many malignant cells overexpress so called multidrug resistant transmembrane proteins which are able to remove the drug from the cell using ATP-dependent active transporter mechanisms. Therefore, resistance to multiple chemotherapeutic agents is a common clinical problem in the treatment of soft tissue sarcomas. HDPs kill malignant cells due to a unique mechanism involving membrane disruption. The fact that there is a difference between membranes of malignant and non-malignant cells makes it possible to develop peptides with selectivity against cancer cells. Whereas normal mammalian cells possess zwitterionic membranes [30], cancer cells have a more negatively charged membrane due to 3–9% phosphatidylserine or glycosaminoglycans [31], [32]. The membranolytic activity of [D]-K_3_H_3_L_9_ was previously analyzed towards model membranes and prostate cancer cells [23]. In this study we detected typical signs of membrane disruption. The premature LDH release gives a hint of disruption of the membrane integrity as a basic cause of the decreased cell vitality. By labeling the peptide via a specific primary antibody we could show a destroyed cell membrane after 4 hours of incubation with a lethal dose of [D]-K_3_H_3_L_9_. These results were further verified by using scanning electron microscopy. The cationic peptide may bind to the anionic structures (e.g. phosphatidylserine) of the malignant cell membrane in a carpet-like manner. After reaching a threshold concentration the peptide may penetrate the membrane leading to a depolarization and death of the cell [11]. Another reason for this selective membrane disruption is the relatively high number of microvilli projections on tumor cells. This leads to a larger surface and to the possibility of higher levels of HDP interaction. Due to the strong membranolytic activity of [D]-K_3_H_3_L_9_ tumor cells are probably not capable to develop resistance. In previous studies bacteria treated with cationic HDPs did not show any resistance against the administered peptides [15], [33].

*In vivo*, two xenograft models were used. Human synovial sarcoma cells were injected into athymic, immune deficient nude mice. Relatively few studies on oncolytic activity of HDPs have been performed in syngeneic models. Immunocompetent mouse models possess the advantage of investigating possible immunomodulatory properties of the peptides. Here the immunocompetent C57BL/6 mice model was treated with syngeneic murine fibrosarcoma cells (BFS-1). BFS-1 cells, originally induced in a female C57BL/6 mouse after treatment with methylcholanthrene, are now able to produce a tumor in an immunocompetent mouse model. Due to its intact immune system this model is closer related to clinical situations. Furthermore the model allows investigating the potential involvement of HDPs in the innate and adaptive immune system.

Here [D]-K_3_H_3_L_9_ could show significant oncolytic activity in both sarcoma xenograft models. Tumors treated with the carrier control PBS show an exponential growth, whereas tumors treated with the [D]-K_3_H_3_L_9_ show partial or in two cases also total remission of the tumor. An antiproliferating activity could be demonstrated in histological and immunohistological samples after treatment with the peptide.

In addition to the potent inhibition of tumor growth the immunohistochemical laminin-staining of the tumors treated with [D]-K_3_H_3_L_9_ revealed a significant decrease in vasculature compared with untreated mice (Fig. 9 (c)). This may be the result of either a reduced cancer cells density, yet unknown, direct vascular targeting of the peptide or even a possible induction of angiogenic inhibiting factors [34]. Soft tissue sarcoma are often markedly angiogenic and highly dependent on their vasculature for primary tumor growth as well as the development of metastases [35]. Until now several antiangiogenic therapies are under evaluation in human STS clinical trials [36], [37]. The convincingly, significant antiangiogenic effect of the peptide [D]-K_3_H_3_L_9_ in this study hold major promise for successful sarcoma therapy but needs further investigation.

On evaluating all the data from killing curves, localization studies, membrane disruption experiments and histopathological studies conducted on living cells under identical experimental conditions, the results suggest a necrotic process. Although a necrotic rather than an apoptotic mechanism of killing is suggested, the details by which [D]-K_3_H_3_L_9_ kills sarcoma cells is still not fully understood. Among the thousands of HDPs isolated thus far, only a few have been investigated for their mode of action on malignant cells [22]. Most of these studies included many biophysical techniques conducted mainly with model phospholipid-membranes [22].

In summary, this study shows that [D]-K_3_H_3_L_9_ can be administered intratumorally, and it dramatically reduces the tumor growth of various sarcoma xenografts. Furthermore, it has an antiangiogenic effect and causes a T-cell attraction in the syngeneic mouse model (Fig. 9 (d+e)), which leads to the assumption of an immunomodulatory effect of the [D]-K_3_H_3_L_9_ peptide. Note also that although peptides can be weakly immunogenic, several reports indicate that free, short HDPs do not induce an antibody response when injected into mice. In addition, the immunogenicity of short fragments containing D-amino acids has been shown to be reduced markedly compared with their all-L- or all-D-amino acid derivates [38], [39]. The unique properties of the diastereomer and its strong membranolytic effect should make it difficult for the tumor cell to develop resistance. Furthermore, designer D-amino acid peptides represent a novel class of oncolytic agents that should be further explored for therapeutic use.
